# Psychometric challenges and proposed solutions when scoring facial emotion expression codes

**DOI:** 10.3758/s13428-013-0421-3

**Published:** 2013-12-06

**Authors:** Sally Olderbak, Andrea Hildebrandt, Thomas Pinkpank, Werner Sommer, Oliver Wilhelm

**Affiliations:** 1 Universität Ulm, Albert-Einstein-Allee 47, 89081 Ulm, Germany; 2 Humboldt-Universität zu Berlin, Berlin, Germany

**Keywords:** Facial emotion expression, Facial action coding system, Automated facial expression recognition, Scoring expression codes, Computer expression recognition toolbox (CERT)

## Abstract

Coding of facial emotion expressions is increasingly performed by automated emotion expression scoring software; however, there is limited discussion on how best to score the resulting codes. We present a discussion of facial emotion expression theories and a review of contemporary emotion expression coding methodology. We highlight methodological challenges pertinent to scoring software-coded facial emotion expression codes and present important psychometric research questions centered on comparing competing scoring procedures of these codes. Then, on the basis of a time series data set collected to assess individual differences in facial emotion expression ability, we derive, apply, and evaluate several statistical procedures, including four scoring methods and four data treatments, to score software-coded emotion expression data. These scoring procedures are illustrated to inform analysis decisions pertaining to the scoring and data treatment of other emotion expression questions and under different experimental circumstances. Overall, we found applying loess smoothing and controlling for baseline facial emotion expression and facial plasticity are recommended methods of data treatment. When scoring facial emotion expression ability, maximum score is preferred. Finally, we discuss the scoring methods and data treatments in the larger context of emotion expression research.

Much about the current state of a person, including level of alertness (e.g., Wierwille & Ellsworth, 1994), direction of attention (Frischen, Bayliss, & Tipper, 2007), and emotional status, is conveyed by facial expressions (Ekman & Friesen, 1982), gaze direction (Frischen et al., 2007), and/or facial flushing (Drummond & Quah, 2001), thus making the face an important component in interpersonal interactions. The ability to effectively *perceive* facial emotional expressions has been intensively studied (e.g., Adolphs, 2006; Heberlein & Atkinson, 2009; Mayer, Salovey, Caruso, & Sitarenios, 2003). However, there is only limited research on the ability to facially *express* one’s emotional state, although this ability is considered a core facet of an individual’s emotional competence (Scherer, 2009).

Traditionally, facial expressions are coded by human raters, but for large amounts of data, this procedure is slow and costly (Ekman & Oster, 1979). Recently, several automated emotion expression software coding programs—for example, the Computer Expression Recognition Toolbox (CERT; Littlewort et al., 2011b) and the FaceReader (den Uyl & Kuilenburg, 2005)—were developed. These programs code the intensity of specific facial muscle movements and/or the intensity of facial emotion expression categories. Arguably, these software programs are at least as precise and reliable as coding by humans (Terzis, Moridis, & Economides, 2010) and may be critical to overcoming several of the limitations associated with human raters.

In addition to the feasibility issues surrounding the *coding* of large amounts of facial expression data, there is the issue of *scoring* these facial expression codes. We define coding as the method for measuring the activation and activation intensity of individual action units and/or facial emotion expression categories, resulting in software-coded facial emotion expression codes, or codes. We define scoring as the method of transforming these codes into *scores* that exhaust the information collected, correspond to what the participants were instructed to achieve, and produce values that are sufficient and efficient for descriptive and inferential statistics. In this article, we will present theories of facial emotion expression, followed by a discussion of contemporary emotion expression coding methodology. We will then discuss methodological challenges pertinent to scoring codes and present important psychometric research questions centered on comparing competing scoring procedures. Then we will derive, apply, and evaluate several statistical procedures for scoring software-coded emotion expression data—specifically, data that were collected to assess individual differences in facial emotion expression ability. Finally, the scoring methods and data treatments will be compared and discussed in the larger context of emotion expression research.

## Theory of facial emotion expressions

Ekman and Friesen (1976) advanced the study of facial emotion expressions by identifying 46 facial action units (AUs). Each AU represents a distinct movement of the face that can occur in isolation from other parts of the face. For example, AU6 identifies the movement known as *cheek raiser* and is based on the activation of the orbicularis oculi, pars orbitalis. The activation of an AU requires the activation of a single facial muscle or a combination of several facial muscles, and the activation of AUs is scored as part of the larger Facial Action Coding System (FACS; Ekman & Friesen, 1978). AUs combine in various configurations to describe a variety of facial expressions—and most often, specific facial *emotion* expressions. These configurations are described in the FACS Affect Interpretive Dictionary, or FACSAID (Ekman, Rosenburg, & Hager, 1998).

The organization and classification of emotions is still intensely debated (for some of the arguments regarding the structure of emotions, see Barrett & Wager, 2006; Ekman, 1992; Izard, 1992; Ortony & Turner, 1990; Russell, 2003; Starkey, 2008); however, for the purposes of this article, we treat emotion as categorical and will focus on six “basic” emotions: anger, disgust, fear, happiness, sadness, and surprise (Ekman, 1992). There is empirical support for these six facial emotion expressions because they are universally recognized across cultures (Ekman et al., 1987), although the recognition of these expressions may vary depending on the sex and age of the observer (Elfenbein & Ambady, 2002; Isaacowitz et al., 2007; Jack, Garrod, Yu, Caldera, & Schyns, 2012) and there are also cultural specificities (Elfenbein & Ambady, 2002). It is also recognized that these do not represent all possible facial expressions that are elicited automatically or intentionally for that emotion. For example, Ekman (1993) identified 60 different anger expressions, which share core properties but, between each other, differ slightly and may indicate differences in the state of the person (“sender”), such as the intensity of the emotion, the spontaneity of the expression, and/or differences in the situation or circumstances that provoked the emotion.

Each basic emotion expression is associated with specific AUs. For example, anger is associated with AU4 (brow lowerer; corrugator supercilii), AU5 (upper lid raiser; levator palpebrae superioris), AU7 (lid tightener; orbicularis oculi, pars palpebralis), AU23 (lip tightener; orbicularis oris), and AU24 (lip pressor; orbicularis oris), either acting separately or in combination. Disgust is also associated with AU4, but also with AU9 (nose wrinkler; levator labii superioris, alaquae nasi), AU10 (upper lip raiser; levator labii superioris), AU15 (lip corner depressor; depressor anguli oris), and AU17 (chin raiser, mentalis), again either separately or in combination (Coan & Gottman, 2007).

One can also show a typical facial emotion expression without feeling that emotion, which is referred to as “faking an emotion” (Gross, 2002). Sometimes the difference between a real and a fake emotion expression is identifiable by the activation timing and duration of specific AUs. For example, a “true” happy expression will show the simultaneous presentation of three AUs: (1) AU6 (defined above), (2) AU7 (lid tightener; orbicularis oculi, pars palebralis, and (3) AU12 (lip corner puller; Zygomaticus major). A “fake” happy expression, in contrast, will show only AU12 or will show AU6 and AU7 later than in a “true” happy expression. This is because AU6 and AU7 are displayed by the action of facial muscles that are not typically under conscious control and, instead, are typically activated only when one truly feels the happy emotion (Ekman, Davidson, & Friesen, 1990; Ekman & Friesen, 1982).

Arguably, particular facial expressions are adaptive under situations where that emotion is activated. For example, the surprise emotion expression involves a widening of the eyes and should be triggered when there is a new and unpredicted stimulus. In that situation, widening the eyes increases the scope of one’s visual field, which is instrumental in quickly visually processing that stimulus, allowing situation-appropriate reactions (e.g., duck and cover; Shariff & Tracy, 2011; Susskind et al., 2008).

## Emotion expression coding methodology

The coding of facial emotion expressions is traditionally performed by human raters. Coding by human raters is done either by FACS-certified experts (e.g., Kohler et al., 2008) or by untrained raters (e.g., Rizzo, Neumann, Enciso, Fidaleo, & Noh, 2001). However, recently, several automated emotion expression coding software programs have been developed that might provide emotion expression codes that are cheaper, quicker, and equivalently reliable, as compared with human raters (Terzis et al., 2010). While the use of untrained human raters does occur, we consider the codes produced under this option less precise than the codes of FACS-certified human raters or automated software. For this reason, in the next section, we will focus only on the coding by FACS-certified human raters and automated software.

### FACS-certified raters

FACS coding requires certified raters who are usually trained at a weeklong workshop (see workshops and courses by the Paul Ekman Group LLC). Raters typically achieve adequate-to-high interrater agreement coding individual AUs, including AU activation and AU intensity, and emotion classifications (Sayette, Cohn, Wertz, Perrott, & Parrott, 2001), but the ratings still vary slightly depending on the rater. FACS-certified ratings are an expensive investment (e.g., expense of the training workshop, hourly reimbursement for raters), and they are much too slow for real-time coding (Ekman & Oster, 1979).

As with other rater-based observational coding methodologies, each study needs a number of practice trials, in order to assess initial interrater agreement, routinely having raters code the same stimuli and recoding by the same rater of previously coded trials to check for drift in codes both between and within raters (Jacobs et al., 1988). FACS-certified raters are trained on a set of individual faces, which means that subsequent codes could vary between raters depending on the set of faces used during training. In addition, in their personal life, each rater has been exposed to a unique number and type of faces, which could further bias their FACS ratings of facial emotion expression categories. This means that each rater may have a bias of unknown magnitude and direction in their FACS ratings.

### Automated software

In order to cope with some of the drawbacks associated with human raters, the coding of AUs is more often performed by automated software programs. In general, these programs calibrate a face image against many other faces taken from established databases (Fasel & Luettin, 2003). The sample specificity of the chosen face databases implies that if the face database and the target face deviate notably from each other (e.g., differing in age or ethnicity), the subsequent emotion codes could be biased. This issue is akin to the bias of human raters discussed above; however, analytic approaches to software-specific bias are easier to investigate and quantify (e.g., Littlewort et al. 2011b).

There are several emotion expression coding software programs available. We will restrict our discussion to CERT, a program that is frequently used; its recently updated version is now referred to as FACET and is available at http://emotient.com/index.php.

CERT codes seven emotions (anger, contempt, disgust, fear, happiness, sadness, surprise) and neutral and provides continuous codes for the individual AUs and *x*- and *y*-coordinates for many parts of the face (e.g., right eye). The software achieves 87 % accuracy for emotion classification and 80 % accuracy for AU activation in adults (Littlewort et al. 2011b) and 79 % accuracy for AU activation in children (Littlewort, Bartlett, Salamanca, & Reilly, 2011). CERT applies a multivariate logistic regression (MLR) classifier, which has been trained on a variety of face data sets, to estimate the proportion to which each emotion is expressed in the face (see Littlewort et al., 2011b, for details). The MLR classification procedure provides proportion estimates for each emotion; this results in codes for all emotions ranging between 0 and 1, and, across all emotions, the codes always sum to 1.0. Because all emotion codes are reported as proportions relative to a total of 1, CERT appears to have linear dependencies between the emotion codes. CERT works especially well if the coded face is displaying only one of its seven emotional or neutral expressions, as compared with a face expressing mixed emotions. High neutral codes indicate low emotion expression, whereas a low neutral score indicates high emotion expression. Currently, most research with CERT is focused on validation of the software (e.g., Gordon, Tanaka, Pierce, & Bartlett, 2011). However, CERT has also been used in studies on other facial expressions, not just those related to emotions, including pain (based on AU codes; Littlewort, Bartlett, & Lee, 2009), level of alertness (indicated by blink rate), and experienced difficulty while watching a lecture (based on indicators for smiling; Bartlett et al., 2010), and has been used to develop a tutoring program based on emotion expression (Cockburn et al., 2008).

CERT produces several codes per picture or video frame. Recordings over a 5-s period with standard video settings (e.g., 25 frames per second) will therefore yield codes for a total of 125 frames per participant. This results in multivariate time series data with codes that are autocorrelated both over time, due to the inertia of face expressions in very brief periods, and between emotions, because many emotions share AUs (e.g., surprise and fear share AUs associated with widening the eyes) or are based on antagonistic AUs (e.g., happiness expression activates AU12, which raises the corners of the mouth, whereas the sadness expression activates AU 15, which lowers the corners of the mouth). In addition, depending on characteristics of the video or image, there may be missing data that cannot be accurately estimated by the software and produce invalid codes. Given this data-analytic context, we will next discuss unique challenges associated with scoring data from automated emotion expression coding software and potential solutions to these challenges.

## Data analytic challenges

### Data cleaning

#### Obscuring objects

Software coding of facial expressions can be perturbed by the presence of objects, such as glasses, hair, scarves, or basically anything that obscures part of the face, and by conditions of the study room, such as poor or inconsistent lighting. Thus, care needs to be taken before collecting emotion expressions in order to prevent the effect of these objects on the data (e.g., asking participants to remove or adjust these objects). While a software program might code the emotion expression of a participant with an artifact present, we recommend that the data associated with these images be completely removed because of unknown biases introduced by those artifacts. At the least, the effects of these artifacts on emotion expressions codes should be assessed.

#### Outliers

Because several emotions are simultaneously coded, a multivariate outlier detection tool, which acknowledges the other emotion codes when identifying outlying values for a particular emotion at that time point, is recommended for outlier detection. Some methods are Cook’s distance (Cook, 1977) and Mahalanobis’s distance (Mahalanobis, 1936), of which there are three types: (1) comparison with the sample mean, (2) comparison with the closest observation, and (3) comparison with every observation. In the case of codes based on videos, the data are also time series. In addition, outlier detection is a unique problem in emotion expression data because some outliers could indicate microexpressions. Microexpressions (referred to as leakage by Ekman & Friesen, 1969; Ekman, Friesen, & O’Sullivan, 1988) are thought to be brief changes in facial expression that occur for fractions of a second (Ekman & Friesen, 2003) when a person is suppressing his or her true emotion or expressing a false emotion and his or her true felt emotion slips out. However, the extent to which microexpressions are a problem may depend on the study.

In general, the identification and treatment of outliers should be uniquely considered for each data set and one’s research question. However, our general recommendation, when one is unconcerned with microexpressions, is to estimate Mahalanobis’s distance by comparing with the sample mean and to set identified outliers to a fixed value, which is the individual mean value plus 3 standard deviations, and to repeat this process until there are no more outliers (Barnett & Lewis, 1978).

#### Missing data

Missing data can occur for several reasons. Briefly, the most common forms are (1) missing completely at random (MCAR), which means that the data are missing due to completely unknown random processes; (2) missing at random (MAR), which means that the missing data can be predicted by other measured variables; and (3) missing not at random (MNAR), which means that the missing data can be predicted, but by an unmeasured variable (Rubin, 1976).

Missing emotion expression data could indicate that there was a problem with the image quality (e.g., poor lighting of part of the face) and, thus, the image needs to be adjusted to be successfully read by the software (type MAR). Image adjustment, however, to only some images and not to others may bias those specific images, because the lighting is improved for some facial features but not all, so a subsequent check of adjusted versus nonadjusted images should be conducted. Second, missing data can be caused by the participant’s head movements limiting the visibility of some facial features (e.g., mouth; type MAR). Third, missing data can be caused by problems of the software in recognizing the face or facial movements because the underlying face model does not fit the face it is trying to read (type MAR). Depending on the nature of missing data and the proportion of missingness, different choices of missing data imputation, or data removal, should be considered.

#### Smoothing

Finally, as with any time series data set, the resulting output may include perturbations, sometimes reflecting noise in the data. A potential solution to the noise is to implement a smoothing algorithm, such as loess smoothing. Loess (short for *local regression* and also referred to as *locally weighted polynomial regression*) works by fitting a polynomial regression to every observation, using observations before and after the data point of interest to predict the new smoothed data point, and is advantageous over other methods, such as low-pass filter methods, because there are no assumptions about the probabilistic structure of the observations. The weighting of neighboring data points is usually implemented by assigning higher weights to more proximal points. The polynomial regression is frequently fitted with weighted least squares (Cleveland, 1979).

Loess smoothing has two parameters that can be adjusted by the researcher to customize the smoothing algorithm to one’s data: (1) *λ*, the degree of the local polynomial, for which typical values are linear (*λ* = 1) and quadratic (*λ* = 2); and (2) α, the smoothing parameter, which represents the breadth of neighboring points included in the estimation, with values ranging between 0 and 1 and higher values causing the resulting estimates to be more similar to neighboring estimates and when visualized, curves in the data appear more smooth (Cleveland, 1993). Three fit indices help one select the best smoothing parameter: (1) the Akaike information criterion (AIC; Akaike, 1973), (2) the bias-corrected AIC (Hurvich, Simonoff, & Tsai, 1998), and (3) generalized cross validation (Wahba, 1983). The potential drawbacks of smoothing algorithms are that they might remove important information, such as peak performances, individual variability in emotion expression, and/or microexpressions, and that, depending on how the smoothing algorithm is applied, the resulting data may be easily influenced by outliers (Cleveland, 1993).

### Controlling for facial plasticity

Software programs apply a face model in the coding of facial emotion expressions. This process assumes that every face is comparable to the general face model and to the face models of the emotion expressions and is capable of achieving perfect emotion expression if given proper instruction and enough time. However, this may not always be true, and there may be individual differences in the basic plasticity of a face. For example, individuals might differ in how high they can pull up the corners of their mouth or in how high they can raise or lower their eyebrows, which will systematically affect their ability to reach high-intensity expressions of particular emotions.

One method for incorporating individual differences in facial plasticity is to include additional trials where participants are asked to activate individual AUs as much as possible, in order to estimate their facial plasticity for that particular AU. Then these AU-specific facial plasticity codes can be partialled from the emotion expression codes and provide more adequate data.

### Controlling for baseline emotion

In addition to individual differences in face plasticity, there may be individual differences in the levels of emotion expressed in one’s baseline (“neutral”) facial expression. We hypothesized that even when a participant does not try to express any emotion but just shows a neutral face, he or she may still be coded with a bit of emotion expression, what we call their *baseline emotion expression*. FACS-certified raters utilize baseline expressions in their emotion expression codes by comparing a participant’s emotionally expressive face with that person’s neutral face (Ekman, Friesen, & Hagar, 2002). In contrast, automated emotion expression coding is based on comparing a target face with a database of faces. Therefore, the software programs do not require a neutral baseline expression for emotion expressions (although Noldus’s FaceReader program [den Uyl & van Kuilenburg, 2005] offers a person-specific calibration option), so the subsequent codes may not be properly calibrated to accommodate that person’s baseline emotion expression. Psychometric scoring procedures based on emotion expressions corrected for baseline emotion expression, in addition to facial plasticity, might be perceived as fairer approaches in measuring emotion expressive abilities.

### Scoring methods

CERT codes are usually analyzed by focusing on the individual AUs (e.g., Wang & Gratch, 2009) and estimating the area under the receiver operating curve for each AU (Bartlett et al., 2008; Vural et al., 2010). Some authors have chosen to dichotomize the data (Terzis et al., 2010); however, this is generally considered an inefficient use of the available data (Cohen, 1983).

In the following part of this article, we compare psychometric scoring options to assess the ability to maximally express a desired target emotion, referred to here as *emotion expression ability*; these scoring procedures are applied to video data, which are thus time series data. To capture expression performance, we begin by comparing four scoring methods: (1) arithmetic mean, (2) geometric mean, (3) average area under the curve (average AUC), and (4) maximum value within each set of time series data. Arithmetic mean is the sum of *n* observations divided by *n*. Geometric mean is calculated by multiplying all *n* observations and then taking the *n*th root of that product and is considered less susceptible to the range of the observations, when compared with the arithmetic mean (McAlister, 1879). AUC can be estimated in a variety of ways, depending on how one interpolates between observed values. We applied the linear trapezoidal method (this draws a straight line between observed *y* values and calculates the area below) to estimate the AUC and averaged these values across observations to estimate average AUC so that the final score was comparable to those of the other scoring methods. While not as accurate as other methods, the trapezoidal methods are preferred because they are straightforward and are especially preferred when the estimate of area is the desired variable and the data between samples has similar structure (Yeh & Kwan, 1978). The maximum value represents the highest value for that emotion across the entire trial.

These scoring methods will be compared across the following data treatment conditions: (1) no cleaning (referred to as *untreated*), (2) smoothing with a loess function, (3) smoothing with a loess function and residualizing baseline emotion, and (4) smoothing with a loess function, residualizing baseline emotion, and residualizing facial plasticity. We will illustrate these scoring methods and data treatments with a large data set, and we will focus on emotion expression codes only from the CERT software.

Our primary research question is specific: What is the best scoring method and data treatment for measuring individual differences in facial emotion expression ability? However, through our illustration of how the scoring methods and data treatments perform under differing conditions and across emotions, we hope that these results can inform analysis decisions pertaining to the scoring and data treatment of other emotion expression questions and under different experimental circumstances.

## Method

### Sample

Our original sample consisted of 284 participants between the ages of 18 and 35 years who lived in the Berlin area; all participants self-identified as Caucasian. Data from 39 participants were discarded due to technical problems during testing and/or insufficient video quality. The final sample size was 245 (50 % females), the mean age was 26.38 years (*SD* = 6.07), and the educational background (assessed as highest educational degree attained) was fairly heterogeneous (29 % without and 58 % with a high school degree, 13 % with academic degrees).

### Procedure

Emotion expression ability was measured in a comprehensive study assessing a series of socio-emotional abilities and cognitive functioning (49 experimental tasks in total), personality facets, and self-reported emotional competence. These assessments were conducted at three consecutive sessions distributed over 5–7 days. Each session lasted about 3 h, including two short breaks. Up to 6 participants were jointly tested. The emotion expression tasks, however, were conducted in groups of 3 participants at most, and participants worked on different computers in separate cubicles. After a demographic questionnaire, the expression tasks were administrated during the first 30 min of the first session. The tasks were programmed in Inquisit 3.2, presented on 17-in. screens with a resolution of 1,680 × 1,050 pixels and a refresh rate of 60 Hz, and were presented in a fixed sequence for all participants.

#### Emotion expression tasks

Participants completed a series of tasks designed to test different aspects of emotion expression ability. For brevity, we will discuss only those tasks that provided data analyzed in this article. Each task was composed of many trials, and on each trial, there was first a 10-s preparation interval where the participant saw the name of the facial movement or the emotion to be produced, followed by a 5-s expression interval during which the facial expression of the participant was recorded. During the 5-s expression interval, participants were asked to complete that task to the best of their ability; it is this 5-s expression interval that we analyzed to assess emotion expression ability. To reduce the interference of artifacts, participants were first asked to remove glasses.

##### Task 1: Calibration (to assess baseline and plasticity)

This was the first task; on the first trial, participants were asked to produce a neutral face to assess their baseline emotion expression. Then, participants were asked to move certain parts of their face in extreme ways to assess facial plasticity. Specifically, the movements were (1) pulling the eyebrows together, (2) raising the eyebrows, (3) wrinkling the nose, (4) widening the nostrils, and (5) raising and (6) lowering the corners of the mouth. Participants completed each movement twice. Together with the baseline trial, there was a total of 13 trials.

##### Task 2: Production

Participants were asked to produce a facial expression corresponding to the emotion label presented on the screen. This was done twice for each of the six basic emotions, resulting in 12 trials; the presentation order of the emotions was randomized and presented in the same order for every participant.

##### Task 3: Calibration without a baseline trial

After all emotion tasks were completed, participants were again asked to complete calibration trials (1) through (6) from task 1 twice to reassess general facial plasticity and changes in facial plasticity over the course of the study. There were 12 trials total.

#### Video data

The faces of all participants were videotaped throughout all emotion expression tasks, including the preparation interval. We used three Panasonic HC-V210EG Camcorders, with 704 × 576 resolution and a capture rate of 25 frames per second. Participants sat approximately 1 m from the camera. Faces were illuminated with two lamps from both sides.

**Fig. 1:**
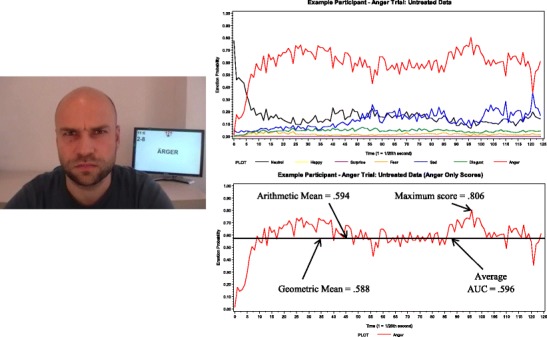
Untreated data. The picture is an example of a participant producing an anger expression. His codes on this trial are displayed in the two right panels. The top right panel includes all emotion codes from an anger trial. The bottom right panel shows only the anger emotion code and illustrates this participant’s anger score as assessed by the four scoring methods

Because the recording of faces was continuous, a first step in the data handling was to parse out the relevant epochs. This was done by including an image trigger shown behind the participants signaling the start and end of each trial (see Fig. 1, left panel). We used Adobe Media Encoder CS4 to parse the video into individual pictures, one picture for each frame. ACDSee Pro 3 and the image trigger were used to identify and select relevant frames from the expression interval of each trial. These frames were then merged into a new video file, with the same resolution settings as before, through VirtualDub v.1.9.11 (www.virtualdub.org), and coded with CERT version 5.1. All CERT codes were analyzed with SAS 9.2.

### Data analytic strategy

The neutral trial from task 1 was used to assess the baseline emotion expression. The calibration trials from both calibration tasks were used to assess facial plasticity. The production trial data were used as the performance measures to compare competing scoring procedures and methods of data treatment. To facilitate our comparison of scoring methods and data treatments, we divided the production trial data into two halves, with one trial for each emotion in each half. Within each half, we estimated every combination of scoring method (e.g., arithmetic mean) and data treatment (e.g., loess smoothed) for each participant and every trial. To illustrate the effect of the scoring methods and data treatments, we will present sample-level means and standard deviations for every emotion trial for every combination of scoring method and data treatment in the first half of the production trials. We will present correlations between the scores from the same scoring method, but generated under different data treatments, with scores from the original untreated data to demonstrate how much participant-level values change as a function of data treatment method. We will also present correlations between values from the first half of the production trials with the corresponding values from the second half of the production trials to test the reliability of these scoring procedures across trials. All of this will be presented separately for each emotion.

### Data cleaning

Before treating and scoring the CERT codes, we first removed all participants with artifacts. Next, we removed all trial-level data where there was more than 20 % missing data for that trial. This concerned 7 participants, with 1 participant completely removed; the remaining 6 participants had, on average, data from 2.7 trials removed. The removed trial-level data were evenly distributed across trials. Finally, the presence of multivariate outliers was tested with Mahalanobis’s distance to the mean. Because the data were time series, outlier detection was conducted within each participant and trial, across time points; we found no statistically significant outliers (Tabachnick & Fidell, 2007).

## Results

### Untreated data

As was described above, CERT provides a continuous emotion code for every video frame and every emotion category (including a neutrality code). Thus, for every facial expression (see Fig. 1) in every frame, CERT will code the relative proportion with which the neutral and seven emotional expressions are expressed. Since we recorded 25 frames per second, one 5-s trial resulted in 125 time points (see top right panel in Fig. 1 for an example of 1 participant’s data for one anger trial).

Because we were interested in the ability to express one specific emotion on each trial, our scoring is based on the target emotion (because CERT emotion codes are linearly dependent, we did not attempt to control for the expression of other possibly related emotions, as suggested by emotion hexagon theory; Calder et al., 1996). For example, on anger trials, participants were asked to produce an angry emotional expression; hence, anger served as the target emotion and was scored for that particular trial (see Fig. 1, right bottom panel). On the basis of a visual inspection of Fig. 1, this participant was able to express anger very well for at least one frame (maximum score = .806); however, across the entire 5-s trial, the participant’s average score was much lower (arithmetic mean = .594; geometric mean = .588; average AUC = .596).

This scoring process was repeated for all production trials. In general, across emotions, scores were highly correlated within a scoring method and between the first and second halves of the production trials (see Correlation with Same Emotion Trial rows in Table 1). Arithmetic mean, geometric mean, and average AUC had the highest average correlations across emotions followed by maximum score. On the basis of these correlations, maximum score appears the least reliable.

**Table 1:** Sample-level values per scoring method across methods of data treatment

		Arithmetic Mean				Geometric Mean				Average AUC				Maximum Score			
Emotion	Sample-Level Scores	Untreated	Loess	Loess + Base	Loess + Base + Plasticity	Untreated	Loess	Loess + Base	Loess + Base + Plasticity	Untreated	Loess	Loess + Base	Loess + Base + Plasticity	Untreated	Loess	Loess + Base	Loess + Base + Plasticity
Anger	*M*	.17	.17	.11	.11	.17	.17	.11	.11	.17	.17	.11	.11	.34	.30	.20	.20
	*SD*	.24	.24	.21	.20	.24	.24	.21	.20	.24	.24	.21	.20	.32	.31	.27	.26
	Correlation with untreated	NA	1.00	.90	.85	NA	1.00	.90	.85	NA	1.00	.90	.85	NA	.99	.87	.83
	Correlation with same emotion trial	.80	.79	.76	.74	.79	.79	.76	.74	.79	.79	.76	.74	.78	.77	.73	.70
Disgust	*M*	.25	.25	.24	.23	.26	.25	.24	.24	.25	.25	.23	.23	.45	.42	.39	.38
	*SD*	.33	.33	.32	.31	.33	.33	.32	.31	.32	.32	.32	.30	.40	.40	.39	.38
	Correlation with untreated	NA	1.00	.98	.94	NA	1.00	.98	.94	NA	1.00	.98	.94	NA	.99	.97	.94
	Correlation with same emotion trial	.70	.70	.68	.66	.69	.69	.68	.66	.70	.70	.68	.66	.60	.63	.61	.58
Fear	*M*	.11	.11	.08	.08	.11	.11	.08	.08	.11	.11	.08	.08	.23	.21	.17	.16
	*SD*	.20	.20	.18	.18	.20	.20	.18	.18	.20	.20	.18	.17	.32	.30	.29	.28
	Correlation with untreated	NA	1.00	.90	.88	NA	1.00	.90	.88	NA	1.00	.89	.88	NA	.99	.94	.92
	Correlation with same emotion trial	.79	.79	.73	.72	.79	.79	.72	.72	.79	.79	.73	.72	.70	.70	.66	.64
Happiness	*M*	.43	.43	.41	.40	.43	.43	.41	.40	.42	.42	.40	.38	.63	.60	.59	.58
	*SD*	.36	.36	.36	.35	.36	.36	.36	.35	.36	.36	.35	.34	.38	.40	.39	.39
	Correlation with untreated	NA	1.00	.99	.97	NA	1.00	.99	.97	NA	1.00	.99	.96	NA	.99	.99	.97
	Correlation with same emotion trial	.82	.83	.83	.82	.83	.83	.83	.82	.83	.83	.82	.82	.78	.79	.79	.78
Sadness	*M*	.31	.31	.20	.19	.31	.31	.20	.19	.30	.30	.20	.19	.49	.44	.30	.29
	*SD*	.29	.29	.25	.25	.29	.29	.25	.25	.29	.29	.25	.25	.32	.33	.29	.28
	Correlation with untreated	NA	1.00	.87	.86	NA	1.00	.87	.86	NA	1.00	.87	.86	NA	.99	.87	.86
	Correlation with same emotion trial	.83	.82	.77	.77	.82	.82	.77	.77	.82	.82	.77	.77	.77	.78	.73	.72
Surprise	*M*	.17	.17	.16	.15	.17	.17	.16	.15	.17	.17	.15	.14	.38	.34	.32	.30
	*SD*	.24	.24	.23	.22	.24	.24	.23	.22	.23	.23	.23	.22	.35	.34	.34	.32
	Correlation with untreated	NA	1.00	.99	.95	NA	1.00	.99	.95	NA	1.00	.99	.95	NA	.99	.98	.94
	Correlation with same emotion trial	.80	.80	.80	.78	.80	.80	.80	.78	.80	.80	.80	.78	.72	.72	.71	.69

*Note*. All correlations were statistically significant at the .05 level. NA = this estimate is not applicable for that particular cell

**Fig. 2:**
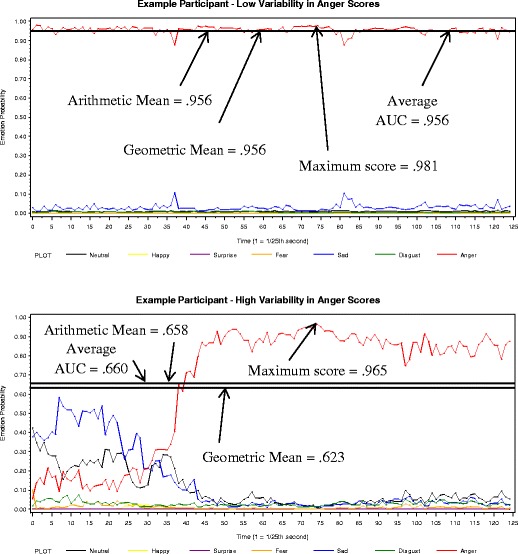
Examples of 2 participants with high and low variability in their anger scores, respectively

Across the trials, arithmetic mean, geometric mean, and average AUC produced very similar scores, and the maximum score was considerably higher than the other scores (see *M* rows in Table 1). This is because arithmetic mean, geometric mean, and average AUC utilize all observations in their analysis, while maximum score is based on a single frame. Although the maximum score appears less reliable across same emotion trials, at this point it appears to be the preferred scoring method. This is because when one’s emotion codes are stable over a trial, there should be minimal differences between the scoring methods (see the top panel in Fig. 2), resulting in all scores being highly correlated. However, if a notable fraction of a participant’s data show momentary peaks in emotion expression, the maximum score will effectively capture that peak in emotion expression, while the other scoring methods will be lower because they incorporate the lower codes as well. In this situation, the correlation between different scoring methods will be lower. The data presented in Table 1 suggest that a considerable portion of the sample showed momentary peaks in their performance, suggesting that participants made a strong effort to produce the target emotion but did not necessarily maintain that peak level throughout the entire trial.

The maximum score, however, needs to be handled with caution because extreme values or potential outliers will more easily affect this scoring method. An inspection of Figs. 1 or 2 shows that CERT will code dramatic (and usually oscillating) changes in emotion expressions between single frames, or for 1/25th of a second. Because these fluctuations arguably reflect coding artifacts, the application of a smoothing algorithm is suggested.

### Smoothed data 

We applied loess smoothing with a quadratic polynomial because this protects against local maxima or minima in our observations (Cleveland, 1993). To identify the best smoothing parameter, we ran a series of loess models, with the smoothing parameter ranging between 0 and 1 by increments of .01, on the target emotion for every trial. We then identified the smoothing parameter associated with the best fit according to each fit index (see Table 2).

**Table 2:** Best smoothing parameter as determined by each fit index

Emotion	Trial	Akaike information criterion		Bias-corrected Akaike information criterion		Generalized cross validation	
		Statistic	Smoothing parameter	Statistic	Smoothing parameter	Statistic	Smoothing parameter
Anger	8	−7.5	.13	−937.9	.13	.000011	.08
	12	−7.5	.12	−932.1	.13	.000010	.10
Disgust	1	−7.8	.10	−972.1	.10	.000011	.07
	9	−8.2	.10	1,017.6	.10	.000009	.07
Fear	4	−10.3	.13	−1,289.8	.13	.000007	.08
	7	−10.0	.12	−1,248.4	.13	.000007	.08
Happiness	6	−6.5	.10	−810.4	.10	.000013	.05
	10	−6.6	.10	−821.6	.10	.000011	.07
Sadness	3	−6.0	.12	−751.4	.13	.000018	.08
	11	−6.2	.12	−775.2	.13	.000016	.08
Surprise	2	−7.7	.12	−965.3	.12	.000011	.07
	5	−8.0	.10	−1,002.1	.12	.000008	.08

**Fig. 3:**
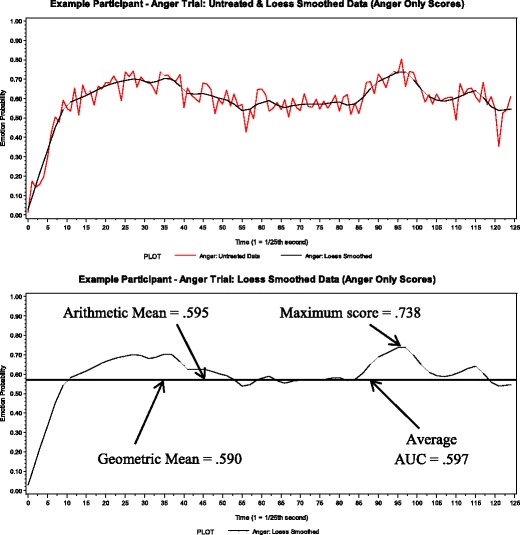
Untreated and loess smoothed anger codes. Top panel illustrates the untreated data with the loess smoothed codes. The bottom panel illustrates only the loess smoothed codes and the four applied scoring methods on the loess data

As can be seen in Table 2, the best smoothing parameters ranged from .10 to .13 for the AIC and for the bias-corrected AIC and from .05 to .10 for the generalized cross validation. Since there was not a single consensus among the fit indices across emotions, we decided to focus on the best smoothing parameters as estimated by the bias-corrected AIC, because this fit index is best suited to protect against overfitting the data (Hurvich et al., 1998). Within the range of best smoothing parameters, .13 occurred the most often, so we decided that the best parameter for smoothing was a quadratic polynomial with a .13 smoothing parameter. Next, these smoothing parameters were applied to all trials of all participants, and each scoring method was computed for the smoothed data (see Fig. 3 for an illustration and Table 1 for sample-level scores).

The sample-level means for arithmetic mean, geometric mean, and average AUC did not change much, as compared with the corresponding sample-level means of the untreated data (see *M* and Correlation with Untreated rows in Table 1), but the sample-level mean for maximum score decreased, suggesting that many of the earlier identified maximum score values reflected some noise or artifact in the data. The correlations between same-emotion trials changed only slightly from the correlations between same-emotion trials with the untreated data.

### Controlling for baseline anger

**Fig. 4:**
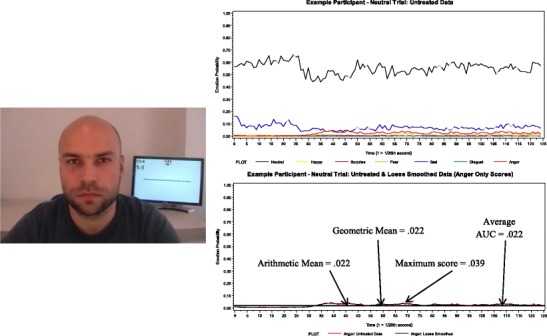
Baseline emotion scores. This picture is an example of a participant displaying a neutral expression. The top right panel illustrates his emotion codes during the course of the neutral trial. The bottom right panel illustrates the process of selecting one emotion, applying the loess smoothing algorithm, and estimating all four scoring methods

Since participants’ ability scores might be biased toward their baseline facial expression, we next controlled for emotion codes estimated during the neutral trial. First, all emotion codes on the baseline trial were smoothed with the settings mentioned previously. Second, each of the different scoring methods was applied; these scores will be referred to as the *baseline scores* (see Fig. 4). Third, the emotion-specific baseline emotion scores were residualized from the respective trial-level target loess smoothed emotion scores in a linear regression. This was done within a scoring method; so, for example, the average AUC baseline anger score was residualized from the average AUC target anger score from the loess smoothed data.

We correlated the baseline emotion scores with the target emotion scores between each scoring method (see Table 3). Anger, fear, and sadness were highly correlated, followed by disgust, happiness, and surprise. The maximum score had the lowest correlations across emotions, as compared with the other scoring methods. Anger, fear, and sadness showed the largest drop in sample-level means and the largest decrease in the correlation with the untreated data set and correlation between same-emotion trials, most notably with the maximum scoring method. These results suggest that it is important to control for baseline emotion, especially for anger, fear, and sadness.

**Table 3:** Correlation of target emotion (loess smoothed data) with baseline emotion (loess smoothed data)

Scoring method	Anger	Disgust	Fear	Happy	Sad	Surprise
Arithmetic mean	.44*	.21*	.44*	.15*	.49*	.13*
Geometric mean	.44*	.21*	.45*	.16*	.50*	.13*
Average AUC	.44*	.21*	.44*	.14*	.49*	.13*
Maximum score	.48*	.22*	.32*	.04	.47*	.10

* *p* < .05

### Controlling for baseline and plasticity

**Fig. 5:**
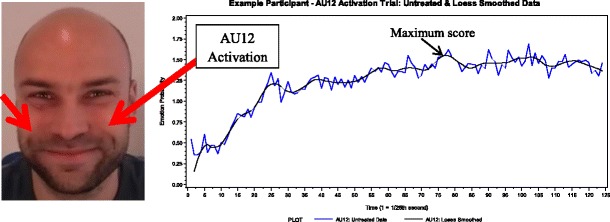
AU12 activation during an AU12 activation trial (calibration trial type 5)

Finally, we controlled for both baseline emotion expression and facial plasticity. The plasticity score was created by working with the AU codes produced on the basis of the calibration trials. On the basis of the instructions of the calibration trials, we selected a corresponding AU (i.e., the one that would be activated specifically during that trial). These were brow lowerer (AU4) for trial type 1, outer brow raiser (AU2) for trial type 2, nose wrinkler (AU9) for trial type 3, lip corner puller (AU12) for trial type 5, and lip corner depressor (AU15) for trial type 6. No single AU was associated with performing trial type 4; therefore, we ignored this trial type in correcting for plasticity. The AU values were first smoothed with the loess smoothing algorithm and settings determined earlier (see Fig. 5 for an illustration). Then, to control for baseline AU activation, the maximum value of the AU was identified on the neutral trial and was subtracted from the respective maximum value AU score from the relevant calibration trial. These difference scores were then *z*-standardized, and a composite score was created for each AU by averaging the respective AU difference score across the relevant calibration trials. Then these composite scores were averaged across AUs to create a single plasticity score.

When controlling for baseline emotion expression and facial plasticity, the sample-level means either dropped a bit further or remained roughly the same, as compared with scores that controlled only for baseline. Also, the correlations between the loess smoothed scores that controlled for baseline and plasticity with the untreated data set between same-emotion trials dropped.

## Summary and recommendations

The above results suggest that sample-level emotion scores will change depending on how the data are treated and scored. As was expected, the highest sample-level mean scores were observed for the maximum value, followed by the arithmetic mean, geometric mean, and average AUC. Those last three scoring methods essentially provided the same sample-level mean and standard deviation scores when compared with each other and across all data treatments, whereas maximum score was affected more by the applied data treatments. We found that smoothing the data had the biggest effect on the maximum value score. Smoothing the data was proposed as a method for dealing with outlier values and noise in the data, which was an effective method for our data, as evidenced by decreasing sample-level maximum value scores. In addition, controlling for baseline emotion expression was important, especially for anger, fear, and sadness. Finally, controlling for facial plasticity showed an additional reduction in sample-level mean values. Thus, the results show that corrections for baseline emotion scores and general face plasticity are likely to allow for psychometrically sound scores. Please note that the loess smoothing settings applied should be carefully considered, since inappropriate settings could drastically distort the data (Cleveland, 1993).

On the basis of data inspection and prior research on the temporal dynamics of facial emotion expressions (Pantic & Patras, 2006; Wehrle, Kaiser, Schmidt, & Scherer, 2000), we have a tentative answer to our research question: Scoring procedures focusing on peak performance are more adequate than procedures summarizing average performance throughout the trial when emotion expression ability is scored. We note that this recommendation might seem somewhat unusual for psychological ability measures, but nevertheless it is the most adequate procedure for indicating the quality of facial emotion expressions. We additionally recommend applying a loess smoothing function and residualizing the baseline emotion and plasticity scores.

Of course, the present recommendations are most applicable to the present tasks and experimental instantiations. Depending on one’s own tasks or instructions, one could possibly want different data treatments or scoring methods. For these reasons, we hope that our illustration of how sample- and participant-level scores changing under different scoring methods and data treatments is helpful.

## Discussion

Automated emotion expression scoring software has many benefits over human raters. However, the data also come with a set of challenges that need to be considered carefully. Some of these challenges also apply to codes by human raters and have not been adequately addressed so far (e.g., determining the best scoring method across time series data), while other challenges are novel (e.g., how to statistically control for facial plasticity). We presented a comparison of four frequently applied scoring methods combined with four data treatments and demonstrated how sample- and participant-level responses change as a function of analytical settings. However, given one’s research question, different approaches to the data may be desirable.

For example, instead of working with the emotion codes, one could work directly with the AU codes. AUs analyzed with multivariate methods, such as network analysis, are useful in identifying patterns in facial expressions and, possibly, testing the validity of emotion-specific expression typologies. Working just with the AUs would also be helpful in identifying differences in the patterns of AU expression that might differ within an emotion-specific expression (e.g., 60 different anger expression patterns; Ekman, 1993). In some cases, these differences in AU activation can help an observer distinguish between a true felt emotion and a fake unfelt emotion (Ekman et al., 1990; Ekman & Friesen, 1982). Another possibility is to further explore the multivariate nature of the expression data, such as examining the likelihood of two emotions being expressed simultaneously, testing the extent to which emotions are expressed separately of other emotions, or testing the extent to which the expression of one emotion reliably predicts the expression of another emotion.

This article presents a comparison of scoring methods and data treatments on CERT codes. We chose CERT for reasons mentioned above. What is further needed in facial emotion expression research is a comparison of available automated emotion expression software coding programs (e.g., FaceReader by den Uyl & Kuilenburg, 2005). In order to better inform researchers working with facial emotion expressions, future research should investigate the similarity and differences between the various available software, including comparisons regarding the classification algorithms, face models, face training databases, and usage characteristics. In addition, while the performance of CERT codes, and other automated emotion expression scoring software, has been compared with the codes by human raters, another interesting direction of research would be to compare the scores generated by automated software with scores generated from facial electromyography (EMG). Preliminary evidence suggests that the CERT codes are comparable with EMG data, even though EMG electrodes are present in the analyzed video (Tanaka, Pierce, Bartlett, Movellan, & Schultz, 2009).

We presented an analysis of CERT codes from an emotion expression task where individuals were asked to explicitly show one emotional expression. The purpose of our task was to look at explicit facial emotion expression ability without any influence of felt emotional valence or imitation of facial expressions. Future research in emotion expression ability should explore other task types, such as imitating facial pictures or perhaps utilizing emotional photos (e.g., International Affective Picture System pictures; Lang, Bradley, & Cuthbert, 1999).

With a tested and recommended method for scoring emotion expression ability, we can now begin to construct more complex measurement models through confirmatory factor analysis in order to understand the structure of emotion expression ability. In addition, we can relate constructs based on emotion expression scores to constructs based on other emotional abilities, such as the ability to perceive and remember faces (Wilhelm et al., 2010) or the ability to perceive and remember emotion in faces (Hildebrandt, Schacht, Sommer, & Wilhelm, 2012).

Through this article, we have illustrated and highlighted some of the many issues with automated emotion expression scoring software programs. While these programs offer many benefits, they also present many new challenges. Through this article, we have tried to highlight some of the specific challenges and illustrate solutions to those challenges. Finally, we have presented suggestions for future lines of research to expand research in this new and interesting field.
